# Systemic metabolic reprogramming within 24 h predicts delayed cerebral ischemia after aneurysmal subarachnoid hemorrhage

**DOI:** 10.3389/fnins.2026.1792615

**Published:** 2026-03-26

**Authors:** Le Guan, Chudi Feng, Xu Wang, Bin Wang, Pan Li, Zhuoyu Yang, Xinyu Zhang, Guoliang Qian, Zigang Yuan

**Affiliations:** 1 Department of Radiology, Shaoxing People's Hospital, Shaoxing, Zhejiang, China; 2 School of Medicine, Shaoxing University, Shaoxing, Zhejiang, China; 3 College of Computer Science and Technology, Changchun University of Science and Technology, Changchun, Jilin, China; 4 Department of Information Centre, Shaoxing People's Hospital, Shaoxing, Zhejiang, China; 5 Department of Neurosurgery, Shaoxing People's Hospital, Shaoxing, Zhejiang, China

**Keywords:** aneurysmal subarachnoid hemorrhage, biomarkers, delayed cerebral ischemia, LC–MS/MS, metabolomics

## Abstract

**Background:**

Delayed cerebral ischemia (DCI) remains a major determinant of poor outcomes after aneurysmal subarachnoid hemorrhage (aSAH), yet early risk stratification is challenging.

**Methods:**

A retrospective cohort of 44 aSAH patients was analyzed (DCI, *n* = 22; non-DCI, *n* = 22). Serum collected within 24 h of admission underwent untargeted liquid chromatography–tandem mass spectrometry metabolomics. Between-group separation was evaluated using supervised multivariate modeling with permutation testing. Differential metabolites were identified using a combined multivariate and univariate strategy (two-sided *P* < 0.05), followed by pathway enrichment analysis. Independent clinical predictors were assessed using multivariate logistic regression, and aneurysm morphology was quantified on admission CT angiography using radiomics-derived parameters.

**Results:**

The DCI group was older, had a higher proportion of females, worse admission neurological status, higher vasospasm incidence, and lower hemoglobin. Vasospasm and age were independent predictors. Aneurysm morphological parameters showed no between-group differences. Metabolic profiles showed clear separation, supported by permutation testing (*R*^2^ = 0.6485; *Q*^2^ intercept = −0.4173). A total of 110 differential metabolites were identified (39 upregulated and 71 downregulated in DCI). Representative changes included increased sphinganine, 2-octenoylcarnitine, guanidoacetic acid, and 5-aminopentanoic acid, with decreased lysophosphatidylcholine 16:0, lysophosphatidylethanolamine 16:0, xanthine, and dehydroepiandrosterone sulfate. Enrichment highlighted coordinated alterations in energy-related, amino-acid, and nucleotide-related pathways.

**Conclusion:**

Early serum metabolomics within 24 h after aSAH revealed a DCI-associated systemic metabolic signature, supporting the identification of exploratory serum metabolic features associated with DCI in a Chinese cohort, which may inform future biomarker development following targeted validation.

## Introduction

Aneurysmal subarachnoid hemorrhage (aSAH) is a devastating subtype of stroke with high mortality and substantial long-term disability (Hoh et al., 2023). Compared with ischemic stroke, aSAH often affects patients at a younger age, resulting in a disproportionate loss of productive life-years and a considerable socioeconomic burden (Lv et al., 2024; Yang et al., 2025). Despite advances in aneurysm occlusion strategies and neurocritical care, many survivors experience secondary neurological deterioration (Hoh et al., 2023; Neifert et al., 2021). Delayed cerebral ischemia (DCI) remains one of the most clinically consequential complications, typically occurring several days after the initial hemorrhage and strongly associated with poor functional recovery (Abdulazim et al., 2023; Vergouwen et al., 2010).

The understanding of DCI has evolved beyond the classic vasospasm-centered model (Chou, 2021). Although angiographic vasospasm is common after aSAH, clinical trials have shown that reducing vasospasm alone does not consistently translate into improved outcomes (Macdonald et al., 2011, 2012). Current evidence supports a multifactorial process involving microcirculatory dysfunction, neuroinflammation, microthrombosis, impaired cerebrovascular autoregulation, and cortical spreading depolarizations (Rowland et al., 2012; Mehra et al., 2023). In routine practice, early identification of patients at high risk for DCI remains challenging, particularly when neurological examination is limited (Abdulazim et al., 2023). This gap underscores the need for accessible, non-invasive biomarkers that can support early risk stratification and guide timely monitoring and intervention.

Metabolomics offers a system-level approach to capture downstream biochemical changes that reflect real-time pathophysiology (Batista et al., 2023). Emerging studies suggest that aSAH is accompanied by widespread metabolic disturbances, including altered energy-related metabolism, shifts in lipid mediators, and changes in amino-acid and nucleotide metabolism (Orban et al., 2024; Lu et al., 2018; Chen et al., 2022b; Yang et al., 2025). These systemic changes may mirror brain–body responses to hemorrhage and may also relate to pathways implicated in secondary injury and inflammation (Gusdon et al., 2022). However, the early peripheral metabolic features that distinguish patients who develop DCI from those who do not remain incompletely defined (Gusdon et al., 2022; Abdulazim et al., 2023).

In this study, we performed untargeted Liquid chromatography–tandem mass spectrometry (LC–MS/MS) metabolomics on serum samples collected within 24 h of admission to characterize early systemic metabolic differences associated with DCI after aSAH. We aimed to identify discriminating metabolites and enriched pathways and to place these findings in the context of clinical risk factors and aneurysm morphology. We hypothesized that early alterations in energy-related metabolism, lipid signaling, and nucleotide turnover would be associated with subsequent DCI. By delineating a serum metabolic signature linked to DCI, this work seeks to support improved prognostication and provide mechanistic clues for future therapeutic exploration.

## Methods

### Study population

This study was approved by the Ethics Committee of Shaoxing People's Hospital and was conducted in accordance with the Declaration of Helsinki. This retrospective study was designed as a case–control analysis. Patients with aSAH admitted to the Department of Neurosurgery between January 2025 and October 2025 were reviewed. Patients who developed DCI were first identified from the institutional database, and an equal number of non-DCI patients were subsequently selected from the same admission period for balanced comparison.

The diagnosis of aSAH was confirmed by non-contrast computed tomography (CT), and the presence of an intracranial aneurysm was verified using CT angiography (CTA) or digital subtraction angiography (DSA). A total of 44 patients were included and divided into the DCI group (*n* = 22) and the non-DCI group (*n* = 22).

DCI was defined according to the multidisciplinary consensus recommendations as: (1) the occurrence of focal neurological impairment (e.g., hemiparesis, aphasia, apraxia, hemianopia, or neglect), or (2) a decrease of at least 2 points on the Glasgow Coma Scale, lasting for at least 1 h, which was not apparent immediately after aneurysm occlusion and could not be attributed to other causes (such as hydrocephalus, rebleeding, infection, or metabolic disturbances) by means of clinical assessment, CT/MRI scanning, and appropriate laboratory studies. Exclusion criteria were as follows: (1) traumatic or mycotic aneurysms, or SAH caused by arteriovenous malformations; (2) history of previous neurological diseases; (3) presence of severe systemic diseases (e.g., malignancy, severe liver or kidney dysfunction, autoimmune diseases) that might significantly affect the serum metabolome; and (4) admission > 24 h after symptom onset.

### CTA-based aneurysm morphological assessment

Admission head and neck CT angiography images were obtained using a 64-detector CT system (Philips Healthcare) with dual-phase bolus injection of 50 mL ioversol followed by 40 mL saline at 4.5–5.0 mL/s. Acquisition parameters were 120 kVp with automatic tube current, a 512 × 512 matrix, 1.0-mm slice thickness, and pitch 0.8. Scanning was triggered at the common carotid bifurcation when attenuation reached 150 HU, and images were subsequently used for post-processing and aneurysm morphological assessment. To minimize measurement bias, an experienced neuroradiologist blinded to the clinical grouping performed the region of interest segmentation and feature extraction.

Based on the reconstructed images, quantitative morphological and density features were extracted to assess their correlation with DCI. Specifically, the maximum diameter was measured as the largest cross-sectional diameter of the aneurysm sac in three-dimensional space, while the neck width was defined as the maximum width of the aneurysm orifice at the level of the parent artery. The height-width ratio was calculated as the ratio of the maximum aneurysm height to the neck width to reflect the geometric aspect ratio. In terms of density features, the maximum Hounsfield unit was recorded as the highest density value within the aneurysm sac region of interest, serving as a surrogate for contrast filling efficiency or potential intra-aneurysmal thrombus. Finally, regarding shape regularity, aneurysms were qualitatively classified as either regular, defined by a smooth surface, or irregular, characterized by the presence of blebs, multiple lobes, or daughter sacs.

### Sample collection and preparation

Peripheral venous blood samples were obtained within 24 h of admission as part of routine clinical care and subsequently retrieved from the institutional biobank for metabolomic profiling. Blood samples were drawn into serum separator tubes. Within 30 min of collection, the samples were centrifuged at 3,000 rpm for 10 min at 4 °C to separate the serum. The supernatant was immediately aliquoted and stored at −80 °C until metabolomic analysis to prevent freeze-thaw cycles.

### Untargeted metabolomics analysis

Serum samples (100 μL) were thawed on ice and mixed with 400 μL of ice-cold methanol to precipitate proteins. The mixture was vortexed and incubated at −20 °C for 30 min. Subsequently, the samples were centrifuged at 20,000 g for 10 min at 4 °C. The supernatant was collected and dried under a gentle stream of nitrogen. Dried samples were reconstituted in acetonitrile:water (1:1, v/v) for LC-MS/MS analysis. A quality control (QC) sample was prepared by pooling equal volumes of all serum samples to monitor system stability. QC samples were generated by mixing equal aliquots of the supernatant from each study sample and were used throughout the analytical workflow to assess signal stability and data quality.

Chromatographic separation was performed on an ACQUITY UPLC HSS T3 column (100 mm × 2.1 mm, 1.8 μm). Mobile phase A consisted of 5 mmol/L ammonium acetate plus 5 mmol/L acetic acid in water, and mobile phase B was acetonitrile. The column temperature was maintained at 40 °C and the injection volume was 4 μL. The flow rate was 0.35 mL/min. The gradient elution program was as follows: 0–0.8 min, 2%−70% B; 0.8–2.8 min, 70%−90% B; 2.8–5.3 min, 90%−99% B; 5.3–5.9 min, 99% B; 5.9–7.5 min, 99%−2% B; 7.5–7.6 min, 2% B; and 7.6–10.0 min, 2% B for re-equilibration.

Mass spectrometric detection was carried out using a Thermo Q Exactive Plus high-resolution tandem mass spectrometer equipped with a heated electrospray ionization source operating in both positive and negative ion modes. Key parameters were: spray voltage, +3.8 kV (positive) / −3.4 kV (negative); capillary temperature, 320 °C. Data were acquired in full scan and data-dependent acquisition modes with a scan range of m/z 70–1,050 and a resolution of 70,000, AGC target of 3E6, and maximum ion injection time of 100 ms. The top 5 precursor ions with intensities greater than 100,000 were selected for MS/MS acquisition, with a DDA resolution of 17,500 and a maximum injection time of 50 ms. Dynamic exclusion was set to 6 s.

Raw LC–MS data were converted to mzXML format and processed in the R environment using XCMS, CAMERA, and the metaX toolbox for peak picking, peak grouping, retention time correction, secondary grouping, and annotation of isotopes and adducts. Each ion feature was defined by retention time and m/z, and a three-dimensional matrix consisting of feature indices, sample identifiers, and ion intensities was generated. Metabolites were annotated by matching exact mass data against the Kyoto Encyclopedia of Genes and Genomes (KEGG) and the Human Metabolome Database (HMDB) databases, with a mass tolerance of 10 ppm; molecular formulas were further supported by isotopic distribution measurements. An in-house MS/MS fragment library was also used to validate metabolite identification. Data preprocessing followed the vendor's standardized workflow in R, including data filtering, missing-value imputation, and normalization. Features were filtered if missing values exceeded 80% across study samples or 50% across QC samples. Remaining missing values were imputed using the k-nearest neighbors method, and data were normalized using probabilistic quotient normalization.

Metabolomics data were processed and analyzed using a standardized untargeted workflow. Group separation was assessed by partial least squares discriminant analysis (PLS-DA), and model validity was evaluated using permutation testing with R2 and Q2 metrics. Differential metabolites between the DCI and non-DCI groups were identified using a combined multivariate and univariate strategy, and were defined by a variable importance in projection (VIP) value > 1 together with a two-sided *P* value < 0.05. The numbers of upregulated and downregulated metabolites were summarized, and results were visualized using a volcano plot and hierarchical clustering heatmap of the top discriminating metabolites. For the eight representative metabolites, group comparisons were performed using log2-transformed normalized intensities. KEGG pathway enrichment analysis was conducted based on the set of differential metabolites, and enrichment results were visualized as a bubble plot. More specifically, PCA and differential metabolite analyses were performed using the R package metaX, hierarchical clustering was visualized using the R package pheatmap, and PLS-DA was conducted using the R package ropls, from which VIP values were derived. KEGG pathway enrichment analysis was performed using a hypergeometric test, and pathways with P < 0.05 were considered significantly enriched.

### Statistical analysis

All tests were two-sided. Continuous variables were first assessed for normality using the Shapiro–Wilk test and were then compared using either the independent-samples Student's *t*-test or the Mann–Whitney *U*-test, as appropriate. Categorical variables were compared using the chi-square test or Fisher's exact test when applicable. Variables significant in univariate analyses were entered into a multivariate logistic regression model, and odds ratios (OR) with 95% confidence intervals (CI) were reported. A *P* value < 0.05 was considered statistically significant. ^*^p < 0.05, ^**^p < 0.01, ^***^p < 0.001, ^****^p < 0.0001.

## Results

### Baseline clinical characteristics and independent predictors of DCI

A total of 44 aSAH patients were included in the final analysis (22 in the DCI group and 22 in the non-DCI group). As detailed in Table 1, patients who developed DCI were significantly older (63.09 ± 13.82 vs. 51.82 ± 15.27 years, *P* = 0.014) and had a higher proportion of females (*P* = 0.005) compared to the non-DCI group. Clinically, the DCI group presented with a more severe initial neurological status, evidenced by lower admission Glasgow Coma Scale (GCS) scores (*P* = 0.021) and higher Hunt-Hess grades (*P* = 0.003). Additionally, the incidence of cerebral vasospasm was markedly higher in the DCI group (54.5% vs. 13.6%, *P* = 0.004). Regarding laboratory findings, admission hemoglobin levels were significantly lower in the DCI group compared to the non-DCI group (114.36 ± 18.19 vs. 127.18 ± 20.24 g/L, *P* = 0.033). This suggests a potential reduction in oxygen-carrying capacity in patients prone to DCI.

**Table 1:** Baseline demographic, clinical, and radiological characteristics of aSAH patients stratified by DCI status.

**Variables**	**Total (*n* = 44)**	**Non-DCI (*n* = 22)**	**DCI (*n* = 22)**	**Statistic**	***P***
Age, Mean ± SD	57.45 ± 15.48	51.82 ± 15.27	63.09 ± 13.82	*t* = −2.57	0.014
Max diameter, Mean ± SD	4.84 ± 2.76	4.72 ± 2.09	4.96 ± 3.35	*t* = −0.29	0.773
Neck width, Mean ± SD	3.00 ± 2.53	2.87 ± 2.07	3.14 ± 2.96	*t* = −0.35	0.726
HWR, Mean ± SD	1.07 ± 0.32	1.04 ± 0.26	1.10 ± 0.38	*t* = −0.62	0.539
Maximum CT value, Mean ± SD	58.68 ± 11.04	56.42 ± 9.29	60.94 ± 12.35	*t* = −1.37	0.178
HGB, Mean ± SD	120.77 ± 20.09	127.18 ± 20.24	114.36 ± 18.19	*t* = 2.21	0.033
DBIL, Mean ± SD	5.91 ± 2.20	6.22 ± 2.54	5.59 ± 1.80	*t* = 0.95	0.347
IBIL, Mean ± SD	9.92 ± 4.06	9.01 ± 3.26	10.82 ± 4.63	*t* = −1.50	0.141
STB, Mean ± SD	15.76 ± 5.53	15.10 ± 5.06	16.41 ± 6.01	*t* = −0.78	0.437
CRP, Mean ± SD	27.83 ± 50.89	27.02 ± 51.16	28.64 ± 51.82	*t* = −0.10	0.918
Neutrophil, Mean ± SD	10.03 ± 10.93	11.94 ± 14.87	8.13 ± 3.95	*t* = 1.16	0.253
Lymphocyte, Mean ± SD	1.36 ± 0.88	1.29 ± 0.58	1.44 ± 1.11	*t* = −0.56	0.578
Monocyte, Mean ± SD	0.79 ± 1.06	1.03 ± 1.45	0.55 ± 0.27	*t* = 1.50	0.140
PLT, Mean ± SD	210.82 ± 74.84	228.09 ± 81.06	193.55 ± 65.36	*t* = 1.56	0.127
NLR, Mean ± SD	10.34 ± 12.05	11.89 ± 15.83	8.80 ± 6.48	*t* = 0.85	0.401
SII, Mean ± SD	2,222.91 ± 3,199.77	2,797.89 ± 4,272.24	1,647.93 ± 1,421.21	*t* = 1.20	0.238
SIRI, Mean ± SD	8.62 ± 15.36	12.28 ± 20.66	4.96 ± 5.29	*t* = 1.61	0.115
GCS, M (Q_1_, Q_3_)	15.00 (9.00, 15.00)	15.00 (15.00, 15.00)	13.00 (8.25, 15.00)	Z=-2.30	0.021
Sex, *n*(%)				χ^2^ = 7.76	0.005
Female	27 (61.36)	9 (40.91)	18 (81.82)		
Male	17 (38.64)	13 (59.09)	4 (18.18)		
Hunt and Hess, *n*(%)				-	0.003
1	15 (34.09)	9 (40.91)	6 (27.27)		
2	13 (29.55)	10 (45.45)	3 (13.64)		
3	12 (27.27)	1 (4.55)	11 (50.00)		
4	4 (9.09)	2 (9.09)	2 (9.09)		
Fisher, *n*(%)				-	0.962
1	8 (18.18)	4 (18.18)	4 (18.18)		
2	24 (54.55)	13 (59.09)	11 (50.00)		
3	4 (9.09)	2 (9.09)	2 (9.09)		
4	8 (18.18)	3 (13.64)	5 (22.73)		
Shape regular, *n*(%)				χ^2^ = 0.07	0.795
Irregular	11 (25.58)	5 (23.81)	6 (27.27)		
Regular	32 (74.42)	16 (76.19)	16 (72.73)		
Vasospasm, *n*(%)				χ^2^ = 8.19	0.004
No	29 (65.91)	19 (86.36)	10 (45.45)		
Yes	15 (34.09)	3 (13.64)	12 (54.55)		

Data are presented as mean ± standard deviation (SD) for normally distributed variables, median (interquartile range, IQR) for non-normally distributed variables, or number (percentage) for categorical variables. P-values were calculated using Student's t-test, Mann-Whitney U test, or Chi-square test, as appropriate.

aSAH, aneurysmal subarachnoid hemorrhage; DCI, delayed cerebral ischemia; HWR, height-width ratio; CT, computed tomography; HGB, hemoglobin; DBIL, direct bilirubin; IBIL, indirect bilirubin; STB, serum total bilirubin; CRP, C-reactive protein; PLT, platelets; NLR, neutrophil-to-lymphocyte ratio; SII, systemic immune-inflammation index; SIRI, systemic inflammation response index; GCS, Glasgow Coma Scale; SD, standard deviation; IQR, interquartile range.

To identify independent risk factors for DCI, a multivariate logistic regression analysis was performed including variables that showed statistical significance in the univariate analysis (age, sex, Hunt-Hess grade, hemoglobin, and cerebral vasospasm). As shown in Table 2, cerebral vasospasm was identified as the strongest independent predictor of DCI (OR 7.16, 95% CI 1.52–33.67, *P* = 0.013). Age was also independently associated with DCI outcomes (OR 1.06, 95% CI 1.01–1.11, *P* = 0.044). Other factors, such as sex and Hunt-Hess grade, were not statistically significant in the multivariate model.

**Table 2:** Univariate and multivariate logistic regression analysis of independent predictors for DCI.

**Variables**	**Univariate analysis**					**Multivariate analysis**				
	***β***	**S.E**	***Z***	***P***	**OR (95%CI)**	***β***	**S.E**	***Z***	***P***	**OR (95%CI)**
**Sex**										
Female					1.00 (Reference)					
Male	−1.87	0.70	−2.66	0.008	0.15 (0.04–0.61)					
**Hunt and Hess**										
1					1.00 (Reference)					
2	−0.80	0.84	−0.95	0.344	0.45 (0.09–2.35)					
3	2.80	1.17	2.40	0.017	16.50 (1.67–163.42)					
15.6-8,-14498pt4	0.41	1.13	0.36	0.720	1.50 (0.16–13.75)					
**Fisher**										
1					1.00 (Reference)					
2	−0.17	0.82	−0.20	0.838	0.85 (0.17–4.20)					
3	−0.00	1.22	−0.00	1.000	1.00 (0.09–11.03)					
15.6-8,-14498pt4	0.51	1.02	0.50	0.615	1.67 (0.23–12.22)					
**Shape regular**										
Irregular					1.00 (Reference)					
Regular	−0.18	0.70	−0.26	0.795	0.83 (0.21–3.29)					
**Vasospasm**										
No					1.00 (Reference)					1.00 (Reference)
Yes	2.03	0.75	2.69	0.007	7.60 (1.73–33.35)	1.97	0.79	2.49	0.013	7.16 (1.52–33.67)
Age	0.06	0.02	2.29	0.022	1.06 (1.01–1.11)	0.05	0.03	2.02	0.044	1.06 (1.01–1.11)
GCS	−0.15	0.09	−1.76	0.079	0.86 (0.72–1.02)					
Longest diameter	0.03	0.11	0.30	0.767	1.03 (0.83–1.28)					
N	0.04	0.12	0.36	0.720	1.05 (0.82–1.33)					
HWR	0.60	0.95	0.63	0.530	1.82 (0.28–11.80)					
Maximum CT value	0.04	0.03	1.35	0.177	1.04 (0.98–1.10)					
HGB	−0.04	0.02	−2.04	0.041	0.96 (0.93–0.99)					
DBIL	−0.14	0.14	−0.95	0.343	0.87 (0.66–1.16)					
IBIL	0.12	0.08	1.45	0.146	1.13 (0.96–1.32)					
STB	0.04	0.06	0.79	0.429	1.05 (0.94–1.17)					
CRP	0.00	0.01	0.11	0.915	1.00 (0.99–1.01)					
Neutrophil	−0.06	0.06	−0.91	0.361	0.94 (0.83–1.07)					
Lymphocyte	0.20	0.35	0.57	0.570	1.22 (0.61–2.45)					
Monocyte	−2.14	1.18	−1.82	0.069	0.12 (0.01–1.18)					
PLT	−0.01	0.00	−1.48	0.138	0.99 (0.98–1.00)					
NLR	−0.03	0.03	−0.80	0.423	0.97 (0.92–1.04)					
SII	−0.00	0.00	−1.03	0.302	1.00 (1.00–1.00)					
SIRI	−0.07	0.05	−1.39	0.166	0.93 (0.84–1.03)					

OR, odds ratio; CI, confidence interval; SE, standard error. Variables with P < 0.1 in the univariate analysis were included in the multivariate model. Bold values indicate statistical significance (P < 0.05).

aSAH, aneurysmal subarachnoid hemorrhage; DCI, delayed cerebral ischemia; OR, odds ratio; CI, confidence interval; N, number; HWR, height-width ratio; CT, computed tomography; HGB, hemoglobin; DBIL, direct bilirubin; IBIL, indirect bilirubin; STB, serum total bilirubin; CRP, C-reactive protein; PLT, platelets; NLR, neutrophil-to-lymphocyte ratio; SII, systemic immune-inflammation index; SIRI, systemic inflammation response index.

### Aneurysm morphological analysis based on radiomics

**Figure 1:**
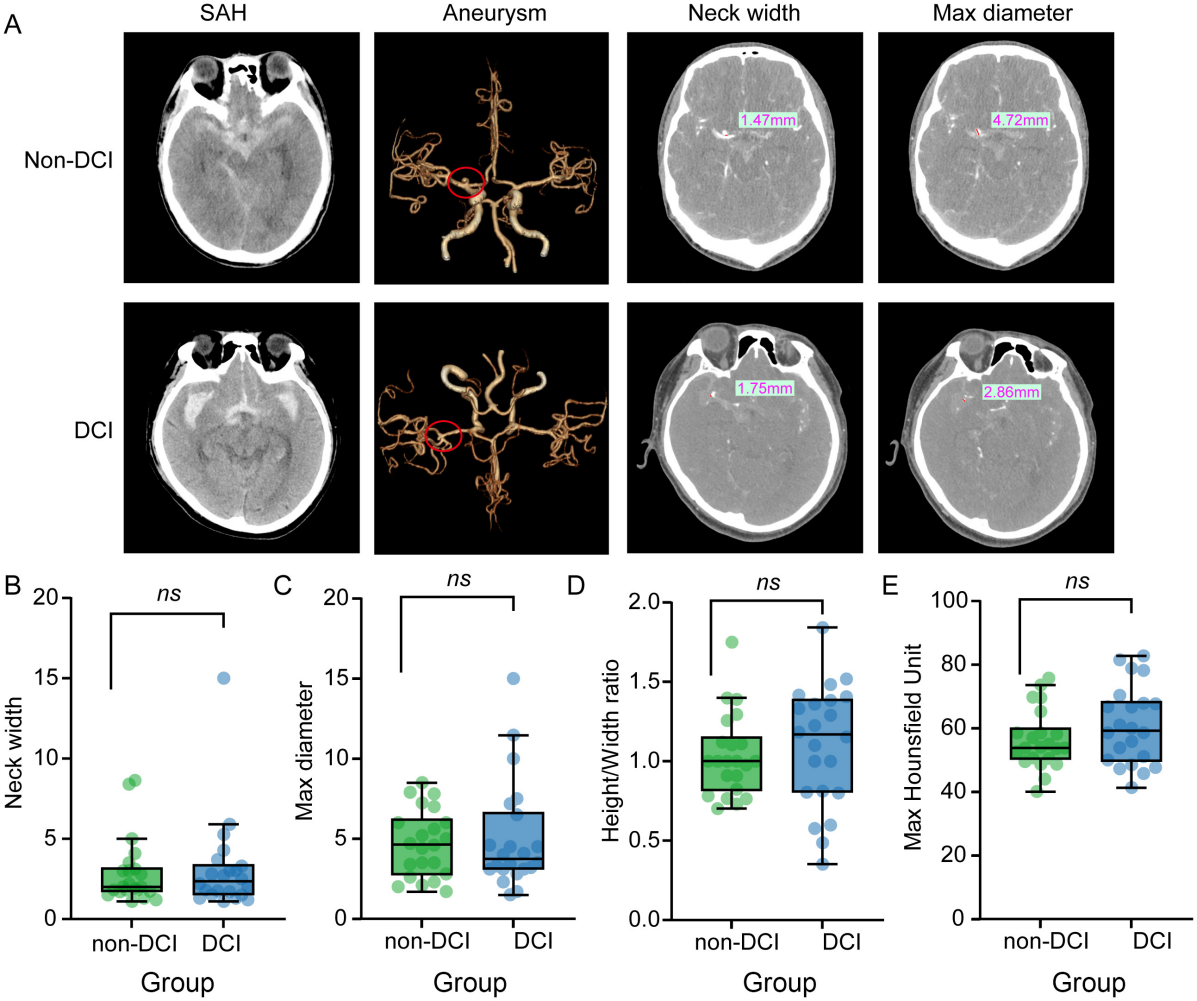
Comparison of aneurysm morphological features between the DCI and non-DCI groups. **(A)** CTA and three-dimensional reconstruction images of intracranial aneurysms from a non-DCI patient (top row) and a DCI patient (bottom row). Measurements of neck width and maximum diameter are illustrated. **(B–E)** Box plots showing the quantitative comparison of morphological parameters: **(B)** Neck width, **(C)** Maximum diameter, **(D)** HWR, and **(E)** HU value. No statistically significant differences were observed between the two groups for any morphological parameters, suggesting that aneurysm anatomy alone does not predict DCI susceptibility in this cohort. CTA, CT angiography; DCI, delayed cerebral ischemia; HWR, height-width ratio; HU, Hounsfield unit; ns, not significant.

Aneurysm morphological features derived from radiomics analysis were further evaluated to determine their potential contribution to DCI susceptibility. Representative CT angiography and 3D reconstruction images of aneurysms are shown in Figure 1A. Quantitative comparison of morphological parameters revealed no statistical differences in Neck width (Figure 1B), Maximum diameter (Figure 1C), Height-Width Ratio (HWR) (Figure 1D), Maximum Hounsfield Unit (Figure 1E), or shape regularity (72.7% vs. 76.2% regular, *P* = 0.795) between the DCI and non-DCI groups (all *P* > 0.05, denoted as ns). This lack of morphological distinction suggests that the susceptibility to DCI in our cohort was not primarily driven by the initial anatomical features of the aneurysm, but potentially by downstream systemic pathophysiological responses.

### Metabolomic profiling and multivariate analysis

**Figure 2:**
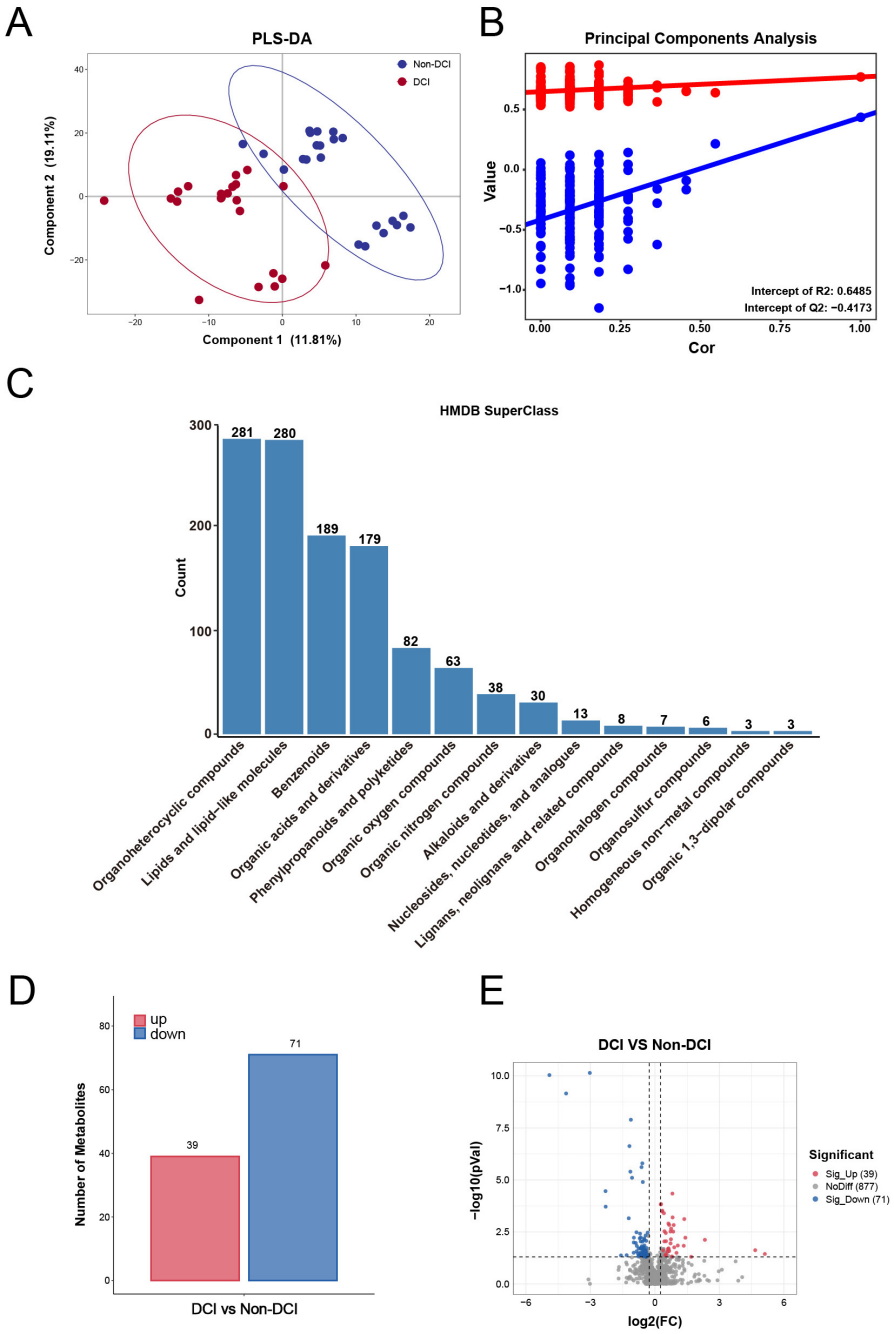
Multivariate statistical analysis and identification of differential metabolites between the DCI and non-DCI groups. **(A)** PLS-DA score plot illustrating the discrimination between the DCI group (red dots) and the non-DCI group (blue dots). The model shows a clear separation between the two groups, with Component 1 explaining 11.81% and Component 2 explaining 19.11% of the variance. **(B)** Permutation test of the PLS-DA model, indicating the robustness and validity of the supervised model (Intercepts: R2 = 0.6485, Q2 = −0.4173). **(C)** Bar chart showing the classification of identified serum metabolites based on the HMDB Super Class. The y-axis represents the number of metabolites in each class. The most abundant classes were “Organoheterocyclic compounds” (*n* = 281) and “Lipids and lipid-like molecules” (*n* = 280), followed by “Benzenoids” (*n* = 189). **(D)** Bar chart summarizing the number of significantly differential metabolites. A total of 110 differential metabolites were identified, with 39 upregulated (red) and 71 downregulated (blue) in the DCI group compared to the non-DCI group. **(E)** Volcano plot visualizing the differential expression of metabolites. The x-axis represents the log2(Fold Change), and the y-axis represents the -log10(*p*-value). Red dots indicate significantly upregulated metabolites, blue dots indicate significantly downregulated metabolites, and gray dots indicate metabolites with no significant difference. Screening criteria: VIP > 1 and *P* < 0.05. DCI, delayed cerebral ischemia; HMDB, Human Metabolome Database; PLS-DA, partial least squares discriminant analysis; VIP, variable importance in projection.

To explore the systemic metabolic alterations associated with DCI, we performed untargeted LC-MS/MS metabolomics on serum samples collected within 24 h of admission. The PLS-DA score plot (Figure 2A) revealed a clear separation between the DCI and non-DCI groups, indicating distinct metabolic phenotypes preceding the clinical onset of ischemia. The permutation test was applied to assess model validity (Figure 2B). The R2 and Q2 intercepts (0.6485 and −0.4173) supported cautious interpretation of the model and did not indicate an obvious overfitting pattern. According to the HMDB classification (Figure 2C), the detected metabolites were predominantly classified as Organoheterocyclic compounds (*n* = 281) and Lipids and lipid-like molecules (*n* = 280), highlighting the extensive involvement of lipid metabolism in the early phase of aSAH.

### Differential metabolite screening and biological significance

**Figure 3:**
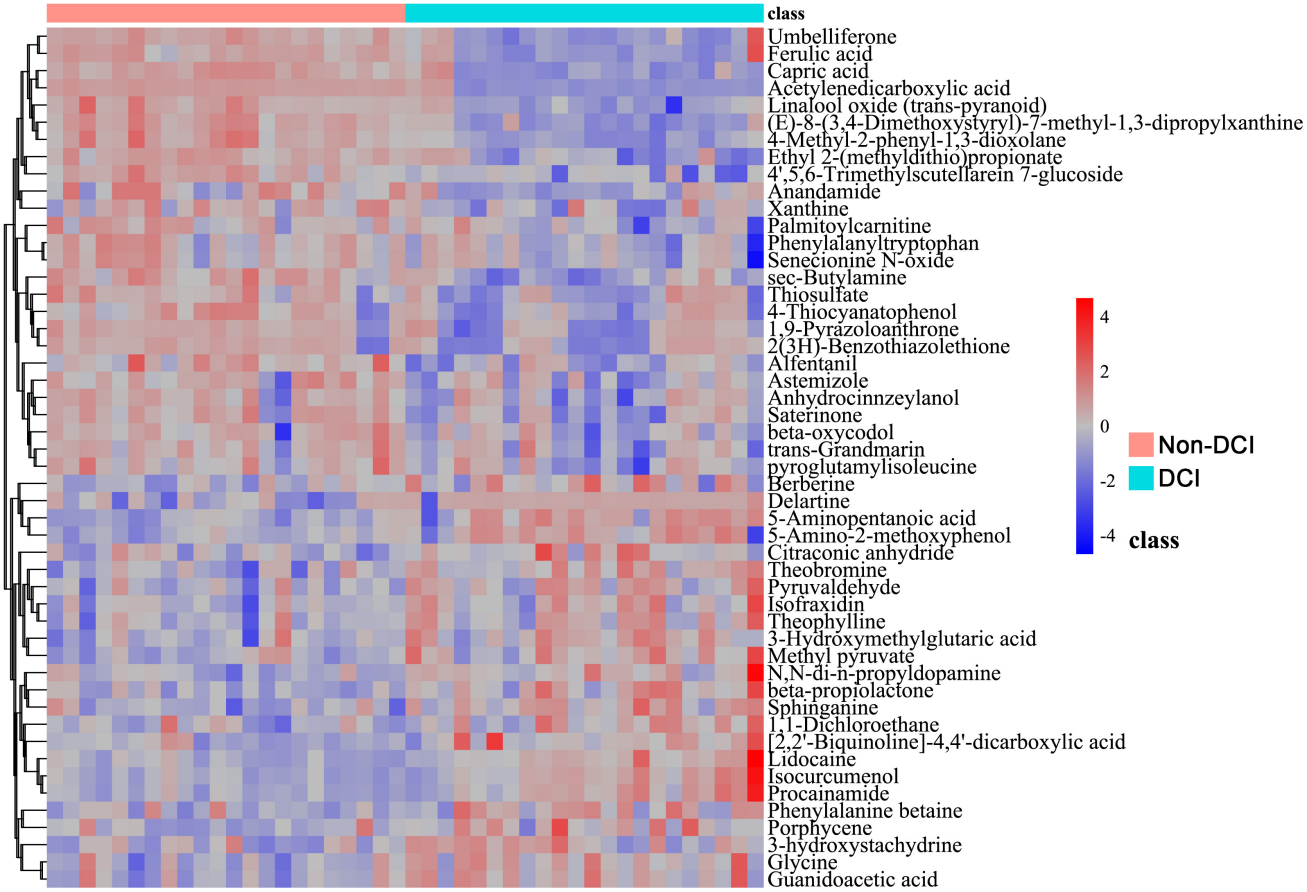
Hierarchical clustering heatmap of differential metabolites. This heatmap visualizes the relative abundance of the top differential metabolites across all study samples. Columns represent individual patients, grouped by clinical outcome (non-DCI group: pink bar; DCI group: cyan bar), and rows represent individual metabolites. The color scale indicates standardized metabolite expression levels, ranging from blue (low abundance) to red (high abundance). The analysis reveals a distinct metabolic signature associated with DCI, characterized by the upregulation of sphingolipid-related metabolites (e.g., Sphinganine) and markers of metabolic stress (e.g., Methyl pyruvate, Glycine, Guanidoacetic acid), alongside the downregulation of acylcarnitines (e.g., Palmitoylcarnitine) and purine metabolites (e.g., Xanthine).

Specific differential metabolites contributing to the separation between groups were further identified. As shown in the bar chart (Figure 2D), 110 metabolites were significantly altered between the DCI and non-DCI groups (VIP > 1, *P* < 0.05), including 39 upregulated and 71 downregulated metabolites in the DCI group. The volcano plot (Figure 2E) depicts the overall distribution of effect sizes and statistical significance, and the hierarchical clustering heatmap (Figure 3) demonstrates a clear group-wise clustering pattern based on the top discriminating metabolites.

**Figure 4:**
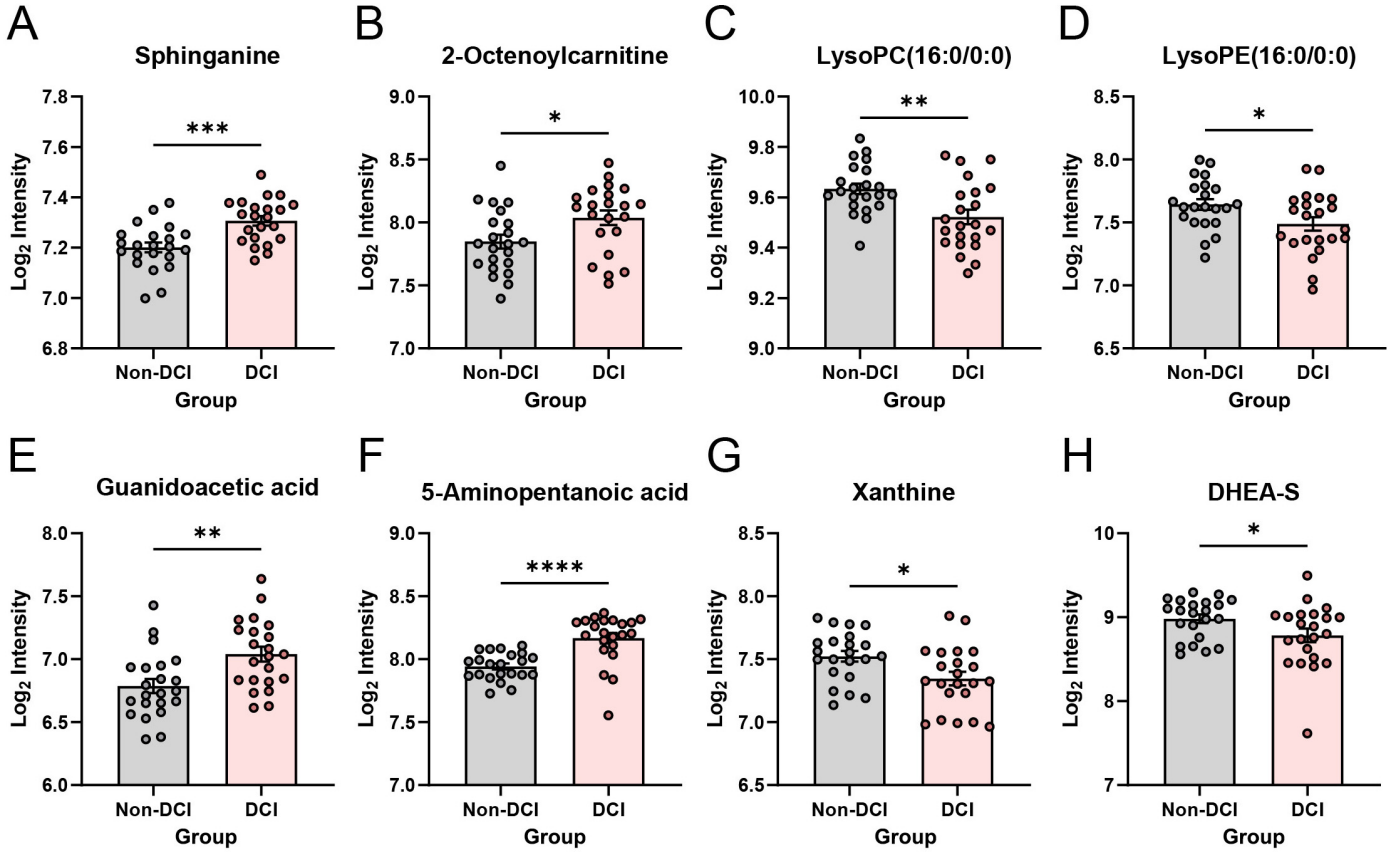
Representative differential metabolites between non-DCI and DCI aSAH patients. Scatter-bar plots show the relative abundance of eight representative metabolites in the non-DCI (*n* = 22) and DCI (*n* = 22) groups. **(A)** Sphinganine. **(B)** 2-Octenoylcarnitine. **(C)** LysoPC (16:0/0:0). **(D)** LysoPE (16:0/0:0). **(E)** Guanidoacetic acid. **(F)** 5-Aminopentanoic acid. **(G)** Xanthine. **(H)** Dehydroepiandrosterone sulfate (DHEA-S). Unpaired t test (or Mann-Whitney test for non-normally distributed variables). Data are expressed as mean ± SEM. *p < 0.05, **p < 0.01, ***p < 0.001, ****p < 0.0001. aSAH, aneurysmal subarachnoid hemorrhage; DCI, delayed cerebral ischemia; LysoPC, lysophosphatidylcholine; LysoPE, lysophosphatidylethanolamine; DHEA-S, dehydroepiandrosterone sulfate.

To further support the differential metabolite signature associated with delayed cerebral ischemia, eight representative metabolites were selected for univariate analysis, using log2-transformed normalized intensities (Figure 4). The DCI group showed higher levels of sphinganine, with a highly significant difference compared with the non-DCI group. 2-octenoylcarnitine was also increased in the DCI group, suggesting altered acylcarnitine dynamics and mitochondrial substrate handling.

In contrast, two lysophospholipids, Lysophosphatidylcholine (LysoPC) (16:0/0:0) and Lysophosphatidylethanolamine (LysoPE) (16:0/0:0), were significantly lower in the DCI group, indicating perturbed membrane lipid remodeling early after aneurysmal subarachnoid hemorrhage. Metabolites related to metabolic stress were concurrently elevated, with guanidoacetic acid and 5-aminopentanoic acid showing robust increases in DCI patients. Conversely, xanthine, a purine catabolite, and Dehydroepiandrosterone sulfate (DHEA-S), a neuroactive steroid, were significantly reduced in the DCI group. Together, these metabolite-level changes highlight coordinated disturbances in lipid signaling, nucleotide turnover, and stress-related metabolism in patients prone to DCI, while xenobiotic or medication-related features detected in the untargeted dataset were not used for mechanistic interpretation.

### KEGG pathway enrichment analysis in DCI

**Figure 5:**
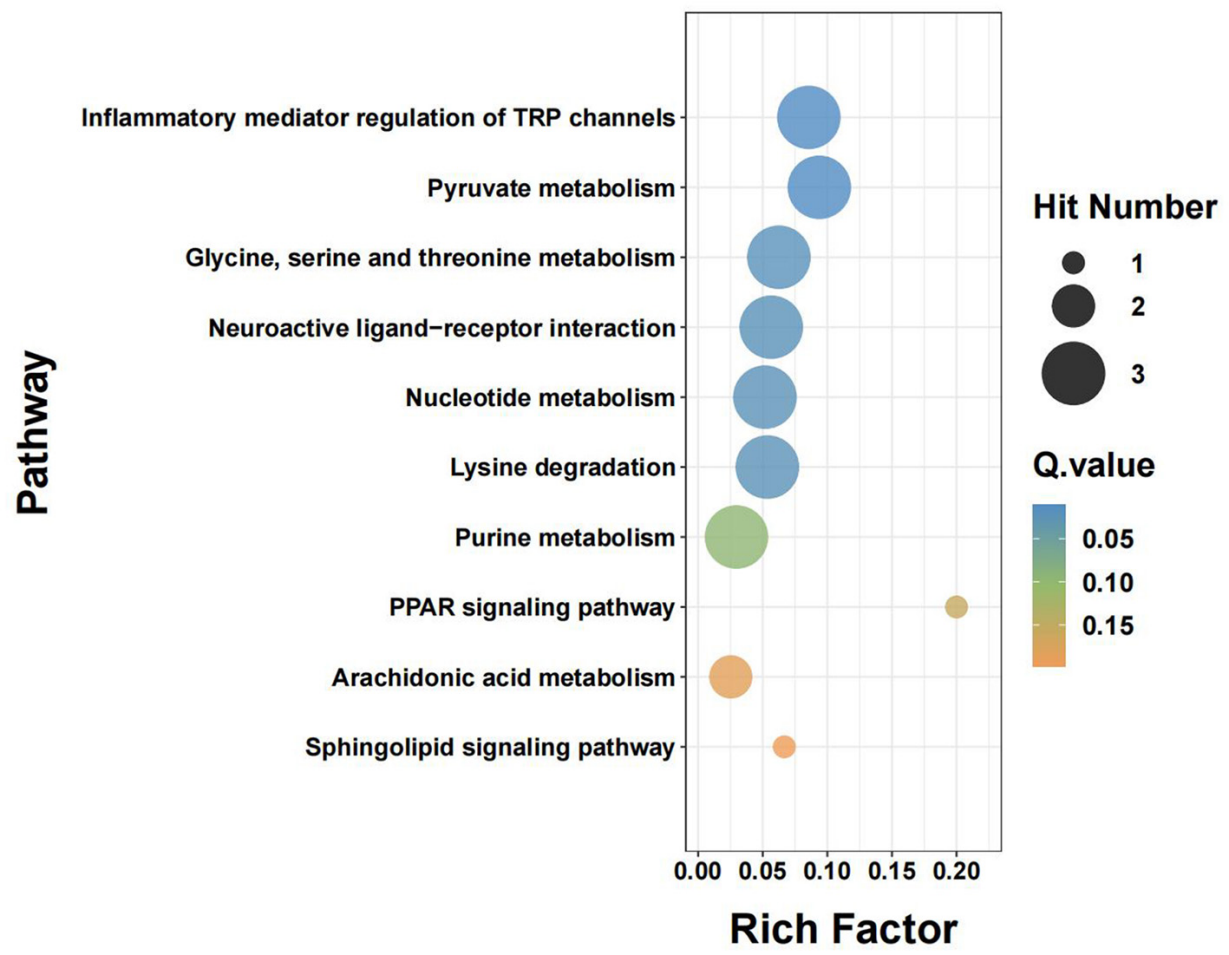
KEGG pathway enrichment scatter plot of DCI-associated metabolites. The bubble plot visualizes the functional distribution of significantly altered metabolic pathways. The X-axis indicates the Rich Factor (ratio of differential metabolites to total metabolites in the pathway), and the Y-axis represents specific KEGG pathways. Bubble size is proportional to the number of metabolites enriched in the pathway (Hit Number). Bubble color represents the statistical significance (Q-value), with cooler colors (blue) indicating higher significance. Pathways such as Pyruvate metabolism and Neuroactive ligand-receptor interaction show the strongest association with DCI, reflecting a systemic transition toward glycolytic metabolism and inflammatory signaling.

To connect the metabolite-level differences with biological function, we carried out KEGG pathway enrichment analysis (Figure 5). The analysis pointed to a broad metabolic shift associated with DCI, spanning energy handling, amino-acid metabolism, nucleotide turnover, and lipid-related inflammatory signaling.

Energy-linked pathways featured prominently, with pyruvate metabolism among the top enriched terms, matching the disturbances in pyruvate-associated intermediates observed in our dataset. Amino-acid pathways were also enriched, particularly lysine degradation and glycine, serine and threonine metabolism. This was in line with the marked increases in 5-aminopentanoic acid and glycine, suggesting a heightened metabolic stress response in patients who developed DCI. In parallel, nucleotide metabolism was enriched, and purine metabolism showed nominal enrichment, consistent with the reduction in xanthine and suggesting altered purine turnover in the DCI group, although the purine pathway did not remain significant after False discovery rate (FDR) correction.

Taken together, the KEGG results indicate that DCI is accompanied by coordinated alterations in bioenergetics, amino-acid metabolism, and nucleotide turnover, together with changes in pathways linked to neuroinflammatory signaling.

## Discussion

This study profiled early serum metabolomic alterations associated with DCI after aSAH using untargeted LC-MS/MS. Using samples collected within 24 h of admission, we identified early metabolic alterations associated with subsequent DCI. We observed group-level metabolic separation between DCI and non-DCI patients in multivariate analysis and identified 110 discriminatory metabolites, together with a representative panel reflecting alterations in lipid remodeling, mitochondrial substrate handling, metabolic stress responses, and nucleotide turnover. Pathway enrichment analysis further highlighted energy-related metabolism and amino-acid and purine pathways. Taken together, these findings suggest that early systemic metabolic vulnerability may be associated with subsequent DCI. However, given the retrospective design, modest sample size, and exploratory nature of untargeted metabolomics, these findings should be interpreted as hypothesis-generating rather than as evidence of causality or definitive biomarker performance.

Conventionally, clinical risk stratification for DCI relies on anatomical and neurological severity scales, such as the modified Fisher and Hunt-Hess grades (Marques et al., 2025). Integrated models like VASOGRADE have further attempted to combine clinical status with admission blood burden (Sharma et al., 2025; Oliveira Souza et al., 2023). More recently, advanced predictive strategies have evolved from CTA-derived morphological analysis to include ultra-early bedside low-field MRI, machine learning-based radiomics, and transcranial Doppler ultrasonography (Yu et al., 2026; Chen et al., 2025; Abdulazim et al., 2023; Chen et al., 2022a). While these tools significantly enhance the sensitivity of ischemia detection, they primarily capture structural or hemodynamic changes that have already begun to manifest. However, as highlighted in recent multidisciplinary reviews, these established tools primarily capture macro-anatomical injury at a single time point and often fail to reflect the dynamic biological susceptibility of the individual patient (Abdulazim et al., 2023; Marques et al., 2025). Consistent with this view, our data suggest that DCI is a multifactorial complication that cannot be fully explained by baseline aneurysm structure or initial hemorrhage burden (Marques et al., 2025; Yu et al., 2026; Dodd et al., 2021). While cerebral vasospasm and age emerged as significant predictors in our multivariable model, neither Fisher grade nor aneurysm morphological parameters distinguished the two groups. These negative findings—potentially influenced by the coarse nature of conventional grading and our modest sample size—reinforce the notion that DCI susceptibility in this cohort is less dependent on baseline anatomical features and more influenced by downstream systemic and pathophysiological responses (Yang et al., 2025). From a translational perspective, this work provides an exploratory framework for identifying candidate peripheral metabolic features, which require targeted quantification and external validation before clinical translation (Jabbarli et al., 2020). Because serum sampling was performed early (within 24 h), these biological readouts could identify high-risk patients who warrant intensified surveillance even before clinical deterioration occurs.

A key finding was the prominent sphingolipid-related signature in the DCI group. The increase in sphinganine may reflect early perturbation of sphingolipid metabolism. Sphingolipids are not merely structural membrane components but are bioactive mediators capable of modulating endothelial function, inflammation, and microvascular tone (Jernigan et al., 2015; Lai et al., 2022; Borodzicz-Jażdżyk et al., 2022; Kerage et al., 2014). In the context of aSAH, where microcirculatory dysfunction and vasospasm are central to DCI pathogenesis, an early shift toward sphingolipid accumulation may reflect membrane stress and lipid signaling programs that favor vascular reactivity and immune activation (Dodd et al., 2021; Ikram et al., 2021; Mehra et al., 2023). In parallel, the significant reduction of lysophospholipids (LysoPC and LysoPE) suggests altered membrane lipid remodeling. Reduced circulating lysophospholipids may indicate accelerated consumption for tissue repair, redistribution into injured neural tissues, or impaired biosynthesis during the acute systemic response (Muralikrishna Adibhatla and Hatcher, 2006; Liu et al., 2017). Together, this specific lipid profile supports a model in which maladaptive membrane remodeling is intrinsically linked to DCI susceptibility.

Beyond lipid remodeling, our data also suggest altered mitochondrial substrate utilization and bioenergetic stress. The elevation of 2-octenoylcarnitine in the DCI group is consistent with disturbed acylcarnitine dynamics, typically signaling incomplete fatty-acid β-oxidation or mitochondrial overload (Schooneman et al., 2013). Notably, our clinical analysis revealed significantly lower admission hemoglobin levels in the DCI group (Shah et al., 2023). This reduced oxygen-carrying capacity may exacerbate systemic hypoxia, thereby contributing to the observed mitochondrial stress signature (Naidech et al., 2007). Such metabolic shifts are biologically plausible, as impaired energy delivery and mitochondrial failure are known drivers of secondary injury cascades after aSAH (Xu et al., 2021).

Metabolites linked to systemic stress were also markedly altered. Guanidoacetic acid and 5-aminopentanoic acid were elevated, aligning with pathway enrichment for lysine degradation and suggesting a hypercatabolic state. Conversely, the depletion of xanthine indicates that purine turnover may also be altered, likely reflecting increased oxidative consumption of purines during ischemic stress (Lalkovičová and Danielisová, 2016). Furthermore, the significantly lower level of DHEA-S in the DCI group is of particular interest. DHEA-S is a neuroactive steroid with anti-inflammatory and neuroprotective properties that naturally declines with age (Maninger et al., 2009; Höllig et al., 2015). Given that our DCI cohort was significantly older and comprised more females, the reduction in DHEA-S may reflect both demographic background and disease-related biological vulnerability (Goldman and Glei, 2007; Labrie, 2010).

Previous metabolomic studies have already compared DCI and non-DCI patients and established early predictive models (Gusdon et al., 2022; Chikh et al., 2024; Yang et al., 2025). Existing literature has reported early disturbances in glycolysis, amino-acid metabolism, and lipid pathways, and demonstrated that metabolite-based models can improve DCI risk prediction (Gusdon et al., 2022; Chikh et al., 2024; Franssen et al., 2025; Yang et al., 2025). Consistent with these findings, the present study also observed early alterations in energy metabolism and amino-acid balance, while further identifying sphingolipid elevation, acylcarnitine abnormalities, and lysophospholipid depletion, suggesting that membrane remodeling and mitochondrial substrate stress may represent additional mechanisms contributing to DCI susceptibility. Differences across studies are likely driven by variability in sampling time points, analytical platforms, clinical endpoints, and population background, as metabolic responses to aSAH may vary across ethnic groups and geographic regions. Collectively, these results support a multidimensional metabolic dysregulation following aSAH, highlighting the need for future multicenter longitudinal studies integrating clinical and metabolomic data to develop robust, population-adapted early prediction strategies for DCI.

This study has several limitations. First, this was a retrospective case–control study with balanced group sizes rather than a consecutive cohort, which may introduce selection bias and limit generalizability. Second, the modest sample size from a single center warrants cautious interpretation and validation in larger cohorts. In addition, all patients were recruited from a single center in China, and thus the identified DCI-associated metabolic signature may be population-specific and requires validation in ethnically diverse cohorts. Third, the absence of healthy controls limits assessment of baseline age- and sex-related metabolic variation, although non-DCI aSAH patients were used as disease-matched controls and age and sex were considered in clinical analyses. Fourth, although we captured early metabolic differences, the observational design does not establish causality; medication exposure and nutritional status may influence serum metabolite levels. Fifth, untargeted annotation requires targeted quantification to confirm absolute concentrations. Finally, the lack of longitudinal sampling prevents the assessment of dynamic metabolic trajectories relative to DCI onset.

## Conclusion

In summary, early serum untargeted metabolomics revealed a distinct systemic metabolic signature associated with subsequent delayed cerebral ischemia after aneurysmal subarachnoid hemorrhage. Patients who developed DCI showed coordinated alterations in lipid remodeling, mitochondrial substrate handling, stress-related amino-acid metabolism, and purine turnover, with pathway enrichment highlighting bioenergetic and catabolic stress programs. These findings suggest that metabolic vulnerability is detectable in peripheral blood within 24 h of admission and may complement clinical risk assessment for early stratification of DCI risk in this Chinese cohort. Further validation in larger and ethnically diverse cohorts, together with targeted quantitative studies, is warranted to support clinical translation.

## Data Availability

The raw data supporting the conclusions of this article will be made available by the authors, without undue reservation.
